# The complete chloroplast genome of *Hemiboea subacaulis* var. *jiangxiensis* Z. Y. Li 1983 (Gesneriaceae), an endemic species in China

**DOI:** 10.1080/23802359.2024.2399929

**Published:** 2024-11-07

**Authors:** Miao Feng, Ji-Si Zhang

**Affiliations:** Liaoning Key Laboratory of Development and Utilization for Natural Products Active Molecules, Anshan Normal University, Anshan, P. R. China

**Keywords:** Chloroplast genome, genetic distance, gesnericaceae, *Hemiboea subacaulis* var. *jiangxiensis*, phylogeny

## Abstract

The complete chloroplast genome of a Chinese endemic species *Hemiboea subacaulis* var. *jiangxiensis* was reported and characterized. The length of chloroplast genome is 153,311 bp, with 37.6% GC content. It has a typical quadripartite structure, including a large single-copy region (LSC, 84,287 bp), a small single-copy region (SSC, 18,094 bp) and a pair of inverted repeats (IRs, 25,465 bp). Totally, 133 unique genes were predicted, including 88 protein-coding genes, 37 tRNA genes and eight rRNA genes. Phylogenetic analysis and genetic distance revealed this species is sister to its typic *H. subacaulis* var. *subacaulis*. This study offers novel genetic data for the molecular identification and phylogenetic analysis of the *Hemiboea*.

## Introduction

The genus *Hemiboea* C. B. Clarke 1888 contains nearly 42 species 5 varieties, and mainly distributed in South China (Li and Wang 2004; Wei 2010; Cui 2023). *Hemiboea subacaulis* Hand.-Mazz. 1925 has two varieties, that is, *Hemiboea subacaulis* var. *subacaulis* Hand.-Mazz. and var. *jiangxiensis* Z. Y. Li. 1983. Both of them are perennial herbs and grow in the subtropical evergreen broad-leaved forests. The former was widely distributed in eastern Guizhou, Hunan, and northern Guangxi, while the latter only distributed in southern Jiangxi (Jinggangshan) (Li and Wang 2004; Wei 2010).

The chloroplast genome of *H. subacaulis* var. *subacaulis* has been published (Cui et al. 2023), and is close related to *H. purpurea* Yan Liu & W. B. Xu 2010. To compare genetic diversity of the two varieties, we sequenced and annotated the complete chloroplast genome of *H. subacaulis* var. *jiangxiensis*, compared genetic distances among *Hemiboea* species, and then reconstructed the phylogenetic relationships within *Hemiboea* to confirm the systematic position of this variety.

## Materials and methods

**Figure 1.**
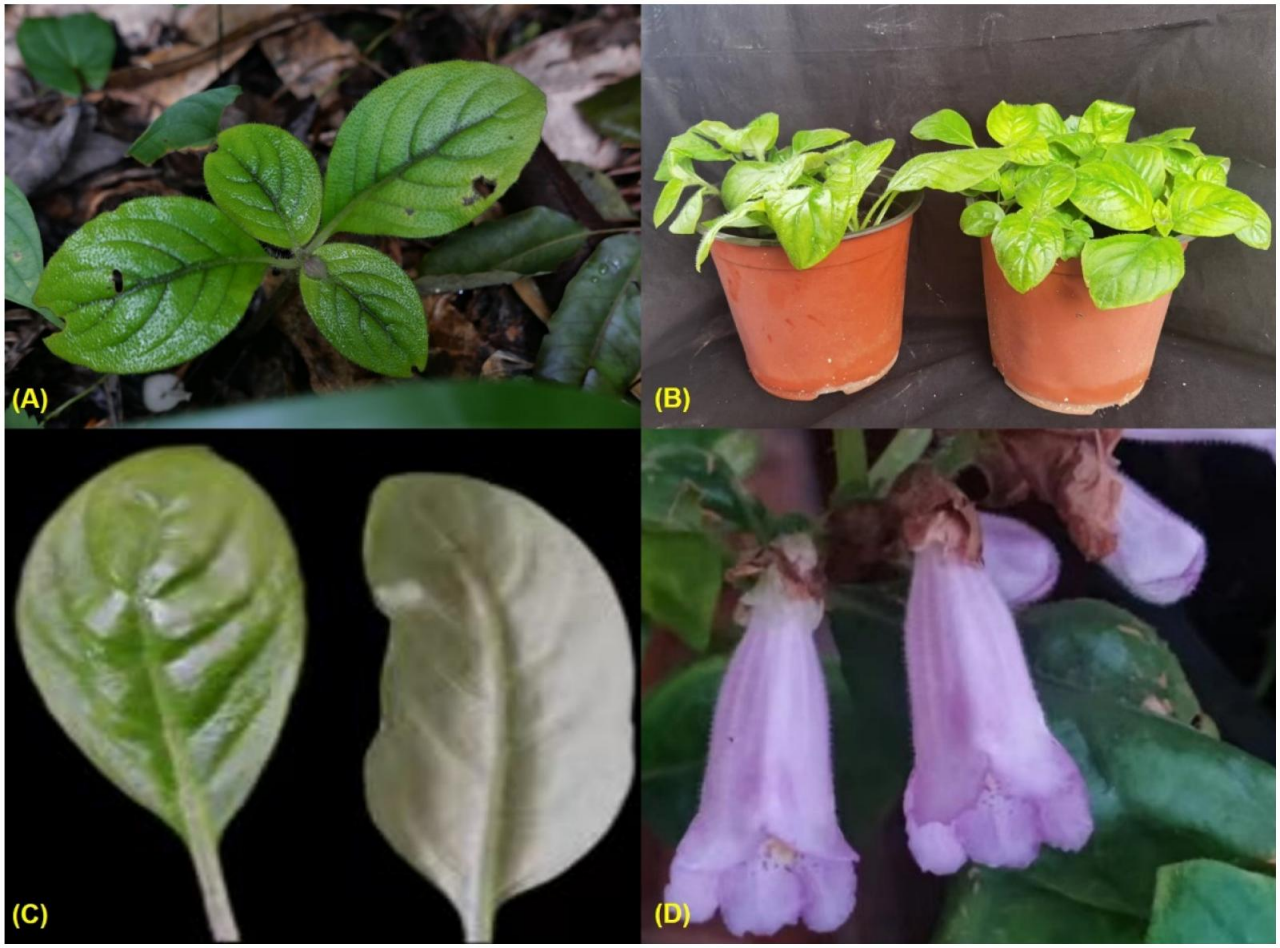
Pictures of *H. subacaulis* var. *jiangxiensis*. (A) habitat (Jinggangshan, Jiangxi Province). (B) cultivated in lab (Anshan Normal University, Liaoning Province). (C) adaxial and abaxial features of leaf. (D) inflorescence. All photos were taken by Jisi Zhang.

Plants of *H. subacaulis* var. *jiangxiensis* were collected in Jinggangshan, Jiangxi, P. R. China (114.170153°N, 26.561692°E, Figure 1A), and cultivated in Anshan Normal University (Figure 1B). The voucher specimen (accession no. ZJS_2023054) was deposited in specimen room of Anshan Normal University (https://www.asnc.edu.cn/, Contact: Jisi Zhang, E-mail: zhangjisi@mail.asnc.edu.cn). This variety is not a protected species, and there is no special permission of fieldwork required.

Total DNA was extracted from fresh leaves using the modified CTAB method (Doyle and Doyle 1987). The library preparation and sequencing using Illumina Hiseq2000 sequencer were conducted at the Kunming Institute of Botany, Chinese Academy of Sciences (Yunnan, China). Generally, four Gb of 150 bp paired-end raw reads were generated for this species. The quality of raw sequence reads was assessed in FastQC v0.11.9 (Brown et al. 2017). The adapters and low quality reads were filtered in Trimmomatic v0.39 (Bolger et al. 2014). The clean reads were assembled using GetOrganelle v1.7.3.2 with default parameters (Jin et al. 2020), and the resulting scaffolds and their connectivity were visualized in Bandage v0.7.1 (Wick et al. 2015). Finally, the plastome was annotated and manually checked in Geneious v9.05 (Kearse et al. 2012) with *Hemiboea ovalifolia* (NC_054358) as a reference. Sequence alignments were performed using MAFFT v7 (Katoh and Standley 2013) and then adjusted manually in Geneious v9.05. The phylogenetic analyses were inferred from the whole plastomes (except one IR) and 80 CDSs for 13 species, respectively (Table S1). The best nucleotide substitution model (TVM + G) for each dataset was calculated using jModelTest2 (Darriba et al. 2012) under the Akaike information criterion. The maximum likelihood (ML) analyses was conducted in RAxML v8.2.12 (Stamatakis 2014) with a rapid bootstrap analysis (1000 replicates) and searching the best-scoring ML tree simultaneously. Each dataset was assigned the GTRGAMMA model due to the model limitation in RAxML. The pairwise K2P (Kimura 2-parameter) distances for the whole plastomes were calculated in MEGA v7 (Kumar et al. 2016).

## Results

**Figure 2.**
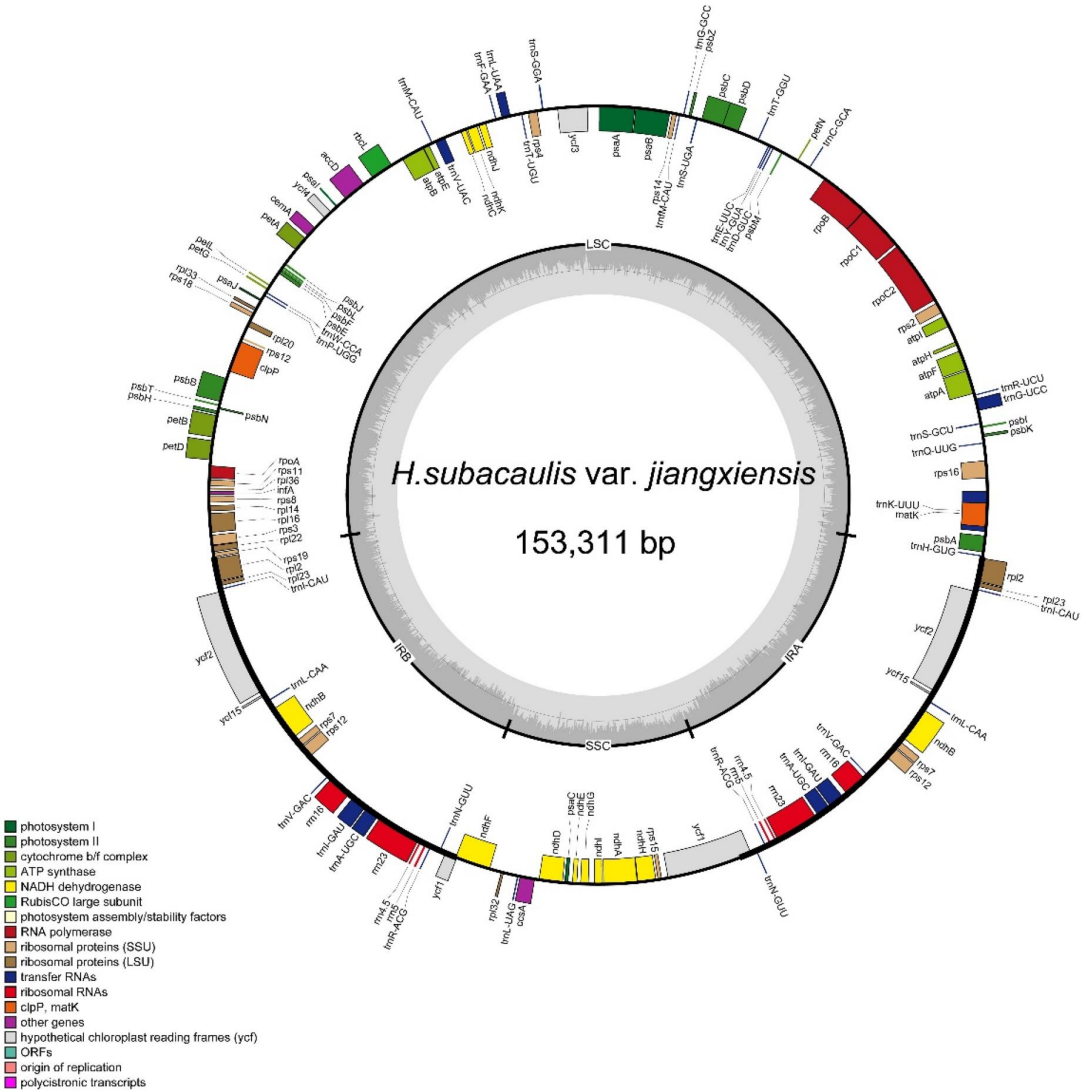
Chloroplast genome map of *H. subacaulis* var. *jiangxiensis*. Different functions groups of genes are signed according to the colored boxes. LSC: large single-copy; SSC: small single-copy; IRA and IRB: inverted repeat regions. The grey circle indicates the GC content.

The plastome size of *H. subacaulis* var. *jiangxiensis* (GenBank: PP816035) is 153,311 bp (Figure 2). Numbers of cis-splicing genes and trans-splicing genes are shown in Supplemental Figures S2 and S3. It contains 84,287 bp large-copy region and 18,094 bp small single-copy region with GC contents of 35.60% and 31.10%, respectively. The length of the two inverted repeat regions is 25,465 bp with GC content of 43.20%. There are 133 genes annotated, comprising 87 protein-coding genes, 37 tRNA genes, 8 rRNA genes and one pseudogens. Among these genes, six CDSs (*ndhB*, *rpl2*, *rpl23*, *rps7*, *ycf15*, and *ycf2*), seven tRNAs (*trnA*-*UGC*, *trnI*-*CAU*, *trnI*-*GAU*, *trnL*-*CAA*, *trnN*-*GUU*, *trnR*-*ACG*, and *trnV*-*GAC*), and four rRNAs (*rrn16*, *rrn23*, *rrn4.5* and *rrn5*) are duplicated in the IR regions. The gene *rps12* contains three exons, two of which are duplicated in the IRs. Further, 16 genes (*ndhA, ndhB, petB, petD, atpF, rpl16, rpl2, rps16, rpoC1*, *trnG*-*UCC*, *trnA*-*UGC*, *trnI*-*GAU*, *trnK*-*UUU*, *trnL*-*UAA*, and *trnV*-*UAC*) have one intron and two genes (*clpP* and *ycf3*) have two introns.

**Figure 3.**
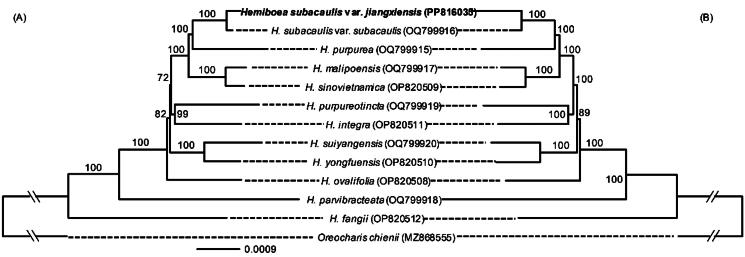
The maximum likelihood (ML) tree of 13 species inferred from 80 CDSs (A) and the complete chloroplast genomes without one IR (B), respectively. Numbers on branches are the supporting values.

The ML tree based on the whole plastomes and 80 CDSs showed similar topologies, except the supporting values of some nodes (Figure 3). The phylogenetic analysis showed that *H. subacaulis* var. *jiangxiensis* and *H. subacaulis* var. *subacaulis* form a clade, and then cluster to *H. purpurea* with strong supporting values (Figure 3). The genetic distance between the two sampled individuals of *H. subacaulis* var. *jiangxiensis* and *H. subacaulis* var. *subacaulis* is 0.001, and the distances between *H. subacaulis* var. *jiangxiensis* and other *Hemiboea* samplings are from 0.003 to 0.008.

## Discussion and conclusion

In this study, the chloroplast genome of *H. subacaulis* var. *jiangxiensis* were newly assembled and annotated. Its chloroplast genome length, GC content and gene composition were similar to other *Hemiboea* species (Cui et al. 2023) and gesnerids (e.g. Hsieh et al. 2022; Wang et al. 2022). The phylogenetic relationships of *Hemiboea* here is similar to results of Cui (2023). Phylogenetic reconstruction revealed that *H. subacaulis* var. *jiangxiensis* is closest to the typical *H. subacaulis* var. *subacaulis* with high supporting values, and the genetic distance between them is much smaller than those among *H. subacaulis* var. *jiangxiensis* and other *Hemiboea* species. Besides, the morphological difference of the two varieties was type of leaf blade, outside pubescent involucre and calyx length. Overall, our study provides valuable genetic data for phylogenetic and evolutionary studies of the genus *Hemiboea*.

Herein, the complete chloroplast genome of *Hemiboea subacaulis* var. *jiangxiensis* is reported for the first. It had a typic quart quadripartite structure, with 153,311 bp in length. Our phylogenetic results showed that the two varieties of *H. subacaulis* formed a clade. The published chloroplast genome of *H. subacaulis* var. *jiangxiensis* will provide genetic information for phylogeny and conservation of *Hemiboea.*

## Supplementary Material

Supporting InformationI.docx

## Data Availability

The genome sequence data that support the findings of this study are openly available in GenBank of NCBI (https://www.ncbi.nlm.nih.gov/) under accession no. PP816035. The associated BioProject, SRA and Bio-Sample numbers are PRJNA1137087, SRR29871207, SAMN42567015.
