# Supplementary material for: The complete chloroplast genome of Hemiboea subacaulis var. jiangxiensis Z. Y. Li 1983 (Gesneriaceae), an endemic species in China
Source: Mitochondrial DNA B Resour. 2024 Nov 7;9(11):1497–500. doi: 10.1080/23802359.2024.2399929 (PMC11544733; doi:10.1080/23802359.2024.2399929)
Supplement: Supporting InformationI.docx [file TMDN_A_2399929_SM6031.docx]

**Supplementary materials**

**Table S1** Taxa, voucher and GenBank accession numbers used in this study.

**Figure S1**. The coverage depth of the chloroplast genome of *Hemiboea subacaulis* var. *jiangxiensis.*

**Figure S2**. Schematic map of cis-splicing genes in the chloroplast genome of *Hemiboea subacaulis* var. *jiangxiensis.*

**Figure S3.** Schematic map of the trans-splicing gene *rps12* in the chloroplast genome of *Hemiboea subacaulis* var. *jiangxiensis.*

**Table S1** Taxa, voucher and GenBank accession numbers used in this study.

| **Taxa** | **Accession No.** | **Source** |
| --- | --- | --- |
| *Hemiboea fangii* | OP820512 | Cui et al., 2023 |
| *Hemiboea integra* | OP820511 | Cui et al., 2023 |
| *Hemiboea malipoensis* | OQ799917 | Cui et al., 2023 |
| *Hemiboea ovalifolia* | OP820508 | Cui et al., 2023 |
| *Hemiboea parvibracteata* | OQ799918 | Cui et al., 2023 |
| *Hemiboea purpurea* | OQ799915 | Cui et al., 2023 |
| *Hemiboea purpureotincta* | OQ799919 | Cui et al., 2023 |
| *Hemiboea sinovietnamica* | OP820509 | Cui et al., 2023 |
| *Hemiboea subacaulis* var. *jiangxiensis* | PP816035 | ZJS_2023054 |
| *Hemiboea subacaulis* var. *subacaulis* | OQ799916 | Cui et al., 2023 |
| *Hemiboea suiyangensis* | OQ799920 | Cui et al., 2023 |
| *Hemiboea yongfuensis* | OP820510 | Cui et al., 2023 |
| *Oreocharis chienii* | MZ868555 | Xu et al., 2022 |

Figure S1. The coverage depth of the chloroplast genome of *Hemiboea subacaulis* var. *jiangxiensis.*


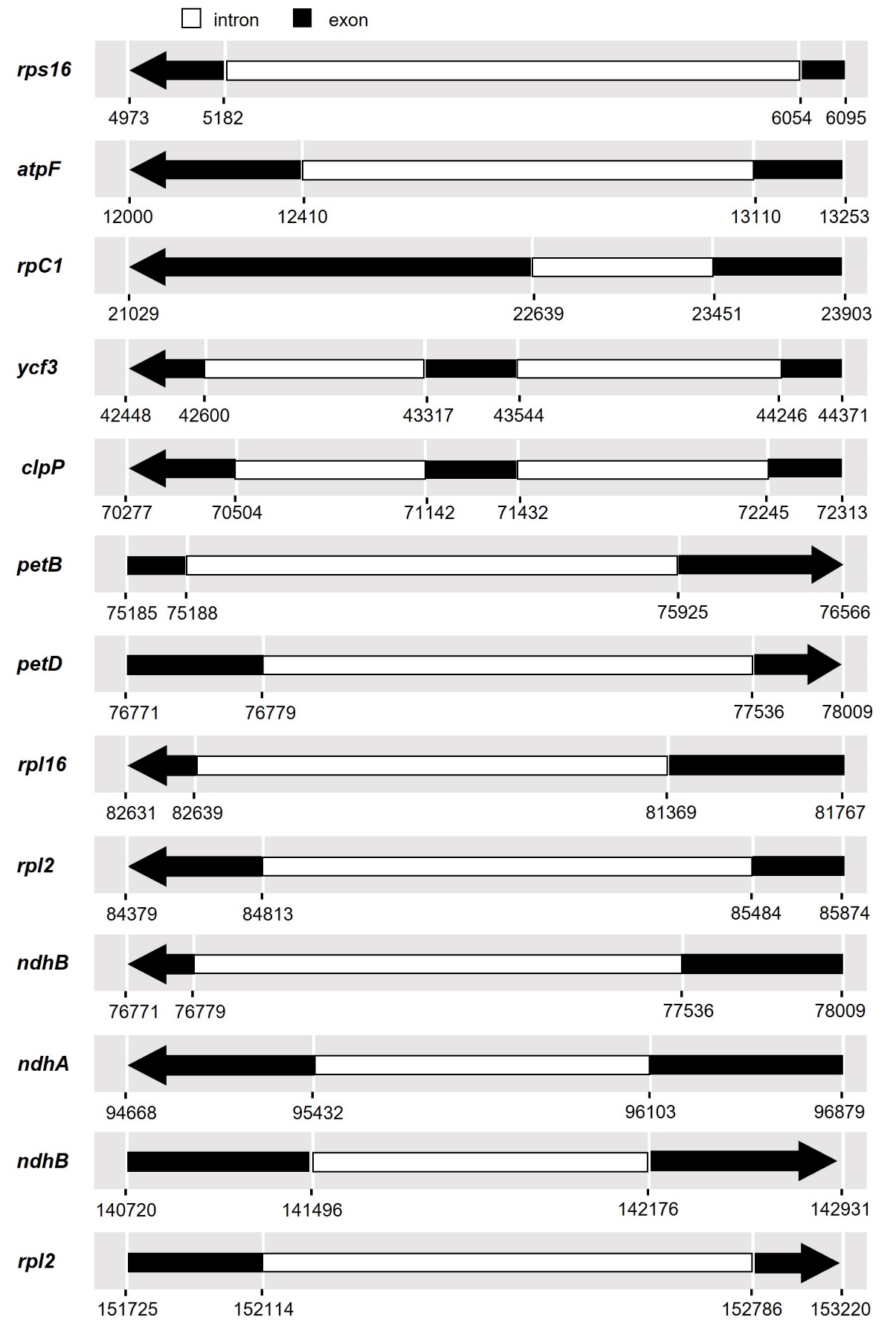


Figure S2 Schematic map of cis-splicing genes in the chloroplast genome of *Hemiboea subacaulis* var. *jiangxiensis.*


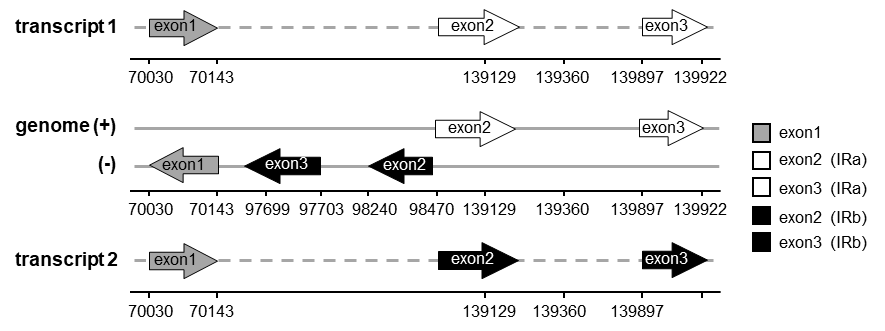


Figure S3. Schematic map of the trans-splicing gene *rps12* in the chloroplast genome of *Hemiboea subacaulis* var. *jiangxiensis.*
